# *Sphingopyxis* Species Isolated from Sand Filter Biofilm at an Australian Drinking Water Treatment Works

**DOI:** 10.1128/mra.00249-23

**Published:** 2023-06-21

**Authors:** Holly-Anne B. Smith, Alex J. Mullins, Gordon Webster, Devin Sapsford, Paul Gaskin, Paul T. Monis, Daniel Hoefel, Christopher P. Saint, Andrew J. Weightman

**Affiliations:** a Microbiomes, Microbes and Informatics Group, Organisms and Environment Division, School of Biosciences, Cardiff University, Cardiff, Wales, United Kingdom; b School of Engineering, Queen’s Buildings, Cardiff University, Cardiff, Wales, United Kingdom; c Dwr Cymru Welsh Water, Coed Kernew, Newport, Wales, United Kingdom; d Australian Water Quality Centre, SA Water, Adelaide, Australia; e Future Industries Institute, University of South Australia, Adelaide, Australia; f UniSA STEM, University of South Australia, Adelaide, Australia; University of Delaware College of Engineering

## Abstract

Three strains isolated by geosmin enrichment from a sand filter in an Australian drinking water treatment works were genome sequenced to identify their taxonomic placement, and a bench-scale batch experiment confirmed their geosmin-degrading capability. Using the average nucleotide identity based on the MUMmer algorithm (ANIm), pairwise digital DNA-DNA hybridization (dDDH), and phylogenomic analyses, the strains were identified as *Sphingopyxis* species.

## ANNOUNCEMENT

Proposed in 2001 (1), the genus *Sphingopyxis* currently comprises 21 validly published species (2), belonging to the family *Sphingomonadaceae* of the class *Alphaproteobacteria*. *Sphingopyxis* strains have been isolated from diverse natural environments, including volcanic rock (3), freshwater (4), soils (5), seawater (6), and some contaminated sites (7–9). *Sphingopyxis* species can utilize a broad range of carbon sources (1), including aromatic compounds such as tetralin (10) and microcystins (11). *Sphingopyxis* species have also been found to biotransform heavy metals and biodegrade polyethers, antibacterials, and geosmin (1, 12).

*Sphingopyxis* strains Geo24, Geo25, and Geo48 were originally isolated in the lab by geosmin enrichment from a sand filter from an Australian drinking water treatment works (13, 14). Geo24 and Geo25 were identified as part of a bacterial consortium able to biodegrade geosmin as the sole carbon source (13), and Geo48 was later identified as an isolate capable of geosmin biodegradation (14). The isolates were stored long term at −80°C, shipped on charcoal transport swabs, streaked onto tryptic soy agar (TSA), and grown for 48 h at 30°C. All strains were streaked three times to ensure the purity of individual colonies.

For genome sequencing, the strains were grown in 5 mL tryptic soy broth (TSB) at 30°C for 48 h, and total DNA was extracted using the FastDNA spin kit for soil (MP Biomedicals). Sequencing was performed on a NovaSeq 6000 SP instrument using a NEBNext Ultra II DNA library prep kit. Between 7 and 9 million (150-bp) read pairs were generated for each genome. The read quality was checked using FastQC v0.11.9, trimming and adapter removal was performed using Fastp v0.20.1, and the genomes were assembled using Unicycler v0.4.7. The genome sizes and other metrics are as follows: Geo24 has a size of 3.86 Mbp, 23 contigs, an *N*_50_ value of 665,169 bp, and a GC content of 65.3%; Geo25 comprises 3.87 Mbp, 23 contigs, an *N*_50_ value of 672,543 bp, and a GC content of 65.3%; and Geo48 has a size of 3.96 Mbp, 25 contigs, an *N*_50_ value of 978,883 bp, and a GC content of 65.2%.

**FIG 1:**
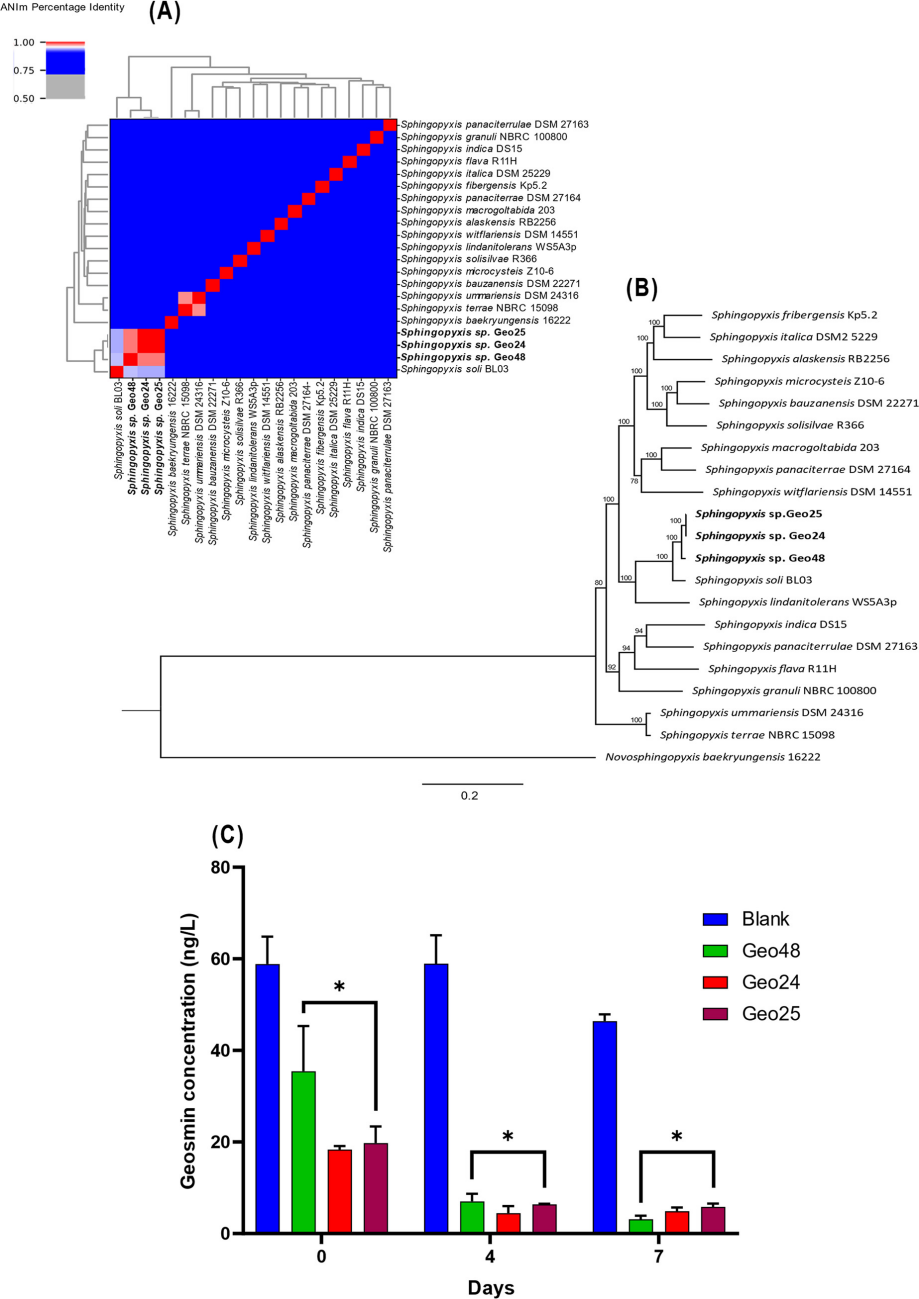
ANIm, phylogeny, and geosmin biodegradation batch experiment results for novel *Sphingopyxis* species Geo24, Geo25, and Geo48, isolated from an Australian drinking water treatment works sand filter. (A) ANIm heatmap produced using PyANI v0.2.12, depicting the average nucleotide identity, as specified by the color bar key. Novel species are shown in bold against *Sphingopyxis* type strains. (B) Phylogenetic tree created using OrthoFinder v2.5.4 and RAxML-NG v1.1 to show the relationship between novel *Sphingopyxis* species and available type strain species, rooted with the type strain from the sister genus *Novosphingopyxis*. The maximum likelihood method was used with the LG model and G4 distribution, with bootstrap support (100 replicates) shown next to each node. Novel *Sphingopyxis* species are shown in bold. (C) Geosmin concentrations for microcosms in batch experiment comparing geosmin removal of novel *Sphingopyxis* species over 7 days. Bacteria (1 mL) grown in TSB, washed, and controlled to an OD_600_ of 1 were added to 9 mL BSM and spiked with 100 ngL^−1^ geosmin. Blank control microcosms with 10 mL BSM, spiked with 100 ngL^−1^ geosmin and with no inoculum, were run simultaneously. Geosmin concentration analysis was performed using SPME with GCMS analysis. Asterisks indicate statistical significance from the blank control at each time point, determined using the Mann-Whitney test.

Phylogenetic analysis of *Sphingopyxis* strains Geo24, Geo25, and Geo48 was performed against all available *Sphingopyxis* type strain genomes, downloaded using the NCBI genome download tool v0.2.10 and annotated using Prokka v1.14.6. The average nucleotide identity (ANI) was calculated using PyANI v0.2.12 (Fig. 1A), and a phylogenomic tree was constructed using OrthoFinder v2.5.4 (15, 16) and RAxML-NG v1.1 (17) (Fig. 1B). All three strains showed an ANI of <94% to the most closely related type strains and displayed phylogenomic distinction, indicating them as potentially novel species (Fig. 1B). Pairwise digital DNA-DNA hybridization (dDDH) values were determined using the Type Strain Genome Server (TYGS) with the most closely related type strains (18); the results indicated that all three *Sphingopyxis* strains represent different species (dDDH, <70%) than those previously described (Table 1).

**TABLE 1:** Basic metrics and pairwise dDDH values for novel *Sphingopyxis* species and the three most closely related type strains^*a*^

Strain	Genome size (Mbp)	GC content (%)	Pairwise dDDH (%) with strain:					
			Geo24	Geo25	Geo48	Sphingopyxis soli BL03	Sphingopyxis lindanitolerans WS5A3p	Sphingopyxis macrogoltabida 203
*Sphingopyxis* sp. Geo24	3.86	65.3		100.0	76.4	50.0	27.5	25.8
*Sphingopyxis* sp. Geo25	3.87	65.3	100.0		76.4	50.0	27.5	25.8
*Sphingopyxis* sp. Geo48	3.96	65.2	76.4	76.4		51.2	27.1	26.2
Sphingopyxis soli BL03	3.63	65.8	50.0	50.0	51.2		27.5	25.9
Sphingopyxis lindanitolerans WS5A3p	4.15	65.3	27.5	27.5	27.1	27.5		25.9
Sphingopyxis macrogoltabida 203	5.75	64.9	25.8	25.8	26.2	25.9	25.9	

a dDDH values were calculated using TYGS and according to the *Sphingopyxis* phylogenetic tree (Fig. 1A). Pairwise dDDH values of <70% indicate different species.

Geosmin biodegradation capacity was confirmed in a microcosm batch experiment by analyzing the geosmin concentration over 7 days. Microcosms comprised of 10 mL bacteria, diluted to 0.1 optical density at 600 nm (OD_600_), in basal salts medium (BSM) (19) in vials with 20 mL headspace. Geosmin losses by volatilization were controlled with microcosms of 10 mL BSM. Geosmin was added at 100 ng/L to each microcosm and measured at 0, 4, and 7 days in triplicate using solid-phase microextraction and gas chromatography mass spectrometry (GCMS) analysis. Significant removal of geosmin was observed for all inoculated microcosms compared to the control (Fig. 1C), demonstrating that all three *Sphingopyxis* strains can degrade geosmin.

### Data availability.

The genome sequences and raw reads have been deposited in the European Nucleotide Archive (ENA) under the project/study number PRJEB60073. The accession numbers for the genome sequences are ERS14837053, ERS14837054, and ERS14837055 for Geo24, Geo25, and Geo48, respectively.

## References

[1] Takeuchi M, Hamana K, Hiraishi A. 2001. Proposal of the genus Sphingomonas sensu stricto, and three new genera, Sphingobium, Novosphingobium and Sphingopyxis, on the basis of phylogenetic and chemotaxonomic analyses. Int J Syst Evol Microbiol 51:1405–1417. doi:10.1099/00207713-51-4-1405. 11491340

[2] Sharma M, Khurana H, Singh DN, Negi RK. 2021. The genus Sphingopyxis: systematics, ecology, and bioremediation potential—a review. J Environ Manage 280:111744. doi:10.1016/j.jenvman.2020.111744. 33280938

[3] Alias-Villegas C, Jurado V, Laiz L, Saiz-Jimenez C. 2013. Sphingopyxis italica sp. nov., isolated from Roman catacombs. Int J Syst Evol Microbiol 63:2565–2569. doi:10.1099/ijs.0.046573-0. 23264504

[4] Baik KS, Choe HN, Park SC, Hwang YM, Kim EM, Park C, Seong CN. 2013. Sphingopyxis rigui sp. nov. and Sphingopyxis wooponensis sp. nov., isolated from wetland freshwater, and emended description of the genus Sphingopyxis. Int J Syst Evol Microbiol 63:1297–1303. doi:10.1099/ijs.0.044057-0. 22798653

[5] Chaudhary DK, Dahal RH, Kim J. 2017. Sphingopyxis solisilvae sp. nov., isolated from forest soil. Int J Syst Evol Microbiol 67:1820–1826. doi:10.1099/ijsem.0.001869. 28613148

[6] Kim B-S, Lim YW, Chun J. 2008. Sphingopyxis marina sp. nov. and Sphingopyxis litoris sp. nov., isolated from seawater. Int J Syst Evol Microbiol 58:2415–2419. doi:10.1099/ijs.0.65614-0. 18842866

[7] Chaudhary DK, Kim J. 2018. Sphingopyxis nepalensis sp. nov., isolated from oil-contaminated soil. Int J Syst Evol Microbiol 68:364–370. doi:10.1099/ijsem.0.002514. 29205125

[8] Kaminski MA, Sobczak A, Spolnik G, Danikiewicz W, Dziembowski A, Lipinski L. 2018. Sphingopyxis lindanitolerans sp. nov. strain WS5A3pT enriched from a pesticide disposal site. Int J Syst Evol Microbiol 68:3935–3941. doi:10.1099/ijsem.0.003094. 30394866

[9] Sharma P, Verma M, Bala K, Nigam A, Lal R. 2010. Sphingopyxis ummariensis sp. nov., isolated from a hexachlorocyclohexane dump site. Int J Syst Evol Microbiol 60:780–784. doi:10.1099/ijs.0.008805-0. 19656938

[10] Ledesma-García L, Sánchez-Azqueta A, Medina M, Reyes-Ramírez F, Santero E. 2016. Redox proteins of hydroxylating bacterial dioxygenases establish a regulatory cascade that prevents gratuitous induction of tetralin biodegradation genes. Sci Rep 6:23848. doi:10.1038/srep23848. 27030382 PMC4814904

[11] Massey IY, Zhang X, Yang F. 2018. Importance of bacterial biodegradation and detoxification processes of microcystins for environmental health. J Toxicol Environ Health B Crit Rev 21:357–369. doi:10.1080/10937404.2018.1532701. 30373489

[12] Yang F, Feng H, Massey IY, Huang F, Guo J, Zhang X. 2020. Genome-wide analysis reveals genetic potential for aromatic compounds biodegradation of Sphingopyxis. Biomed Res Int 2020:5849123. doi:10.1155/2020/5849123. 32596333 PMC7273453

[13] Hoefel D, Ho W, Aunkofer W, Monis PT, Keegan A, Newcombe G, Saint CP. 2006. Cooperative biodegradation of geosmin by a consortium comprising three Gram-negative bacteria isolated from the biofilm of a sand filter column. Lett Appl Microbiol 43:417–423. doi:10.1111/j.1472-765X.2006.01974.x. 16965373

[14] Hoefel D, Ho L, Monis PT, Newcombe G, Saint CP. 2009. Biodegradation of geosmin by a novel Gram-negative bacterium; isolation, phylogenetic characterisation and degradation rate determination. Water Res 43:2927–2935. doi:10.1016/j.watres.2009.04.005. 19439338

[15] Emms DM, Kelly S. 2017. STRIDE: species tree root inference from gene duplication events. Mol Biol Evol 34:3267–3278. doi:10.1093/molbev/msx259. 29029342 PMC5850722

[16] Emms DM, Kelly S. 2018. STAG: species tree inference from all genes. bioRxiv. doi:10.1101/267914.

[17] Kozlov AM, Darriba D, Flouri T, Morel B, Stamatakis A. 2019. RAxML-NG: a fast, scalable, and user-friendly tool for maximum likelihood phylogenetic inference. Bioinformatics 35:4453–4455. doi:10.1093/bioinformatics/btz305. 31070718 PMC6821337

[18] Meier-Kolthoff JP, Göker M. 2019. TYGS is an automated high-throughput platform for state-of-the-art genome-based taxonomy. Nat Commun 10:2182. doi:10.1038/s41467-019-10210-3. 31097708 PMC6522516

[19] Webster G, Jones C, Mullins AJ, Mahenthiralingam E. 2020. A rapid screening method for the detection of specialised metabolites from bacteria: induction and suppression of metabolites from Burkholderia species. J Microbiol Methods 178:106057. doi:10.1016/j.mimet.2020.106057. 32941961 PMC7684528

